# DFT and dynamic simulations of OleuoPectin structures as a potential anticoagulant agent

**DOI:** 10.1038/s41598-025-06000-1

**Published:** 2025-06-20

**Authors:** Mona T. M. Ghanem, Heba D. Hassanein, Ahmed F. El-Sayed, Medhat A. Ibrahim, Ahmed M. Bayoumy

**Affiliations:** 1 Chemistry of Medicinal Plant Department, Pharmaceutical and Drug Industries Research Institute, National Research Centre, Dokki, Giza Egypt; 2 Microbial Genetics Department, Biotechnology Research Institute, National Research Centre, Giza, Egypt; 3 Egypt Center for Research and Regenerative Medicine (ECRRM), Cairo, Egypt; 4 Molecular Modeling and Spectroscopy Laboratory, Centre of Excellence for Advanced Science, National Research Centre, 33 El-Bohouth St, Dokki, Giza 12622 Egypt; 5 Spectroscopy Department, National Research Centre, 33 El-Bohouth St., Dokki, Giza 12622 Egypt; 6 Biophysics Group, Physics Department, Faculty of Science, Ain Shams University, Cairo, 11566 Egypt

**Keywords:** Oleuropein, MESP maps, QSAR, Dynamic simulation, Anticoagulant, Drug discovery, Molecular biology, Mathematics and computing

## Abstract

Oleuropein, a biologically active compound with variable pharmacological properties, is poorly absorbed in the gut, causing gastrointestinal upset in sensitive individuals. Model molecules of Oleuropein, pectin, and some proposed interactions were built up and geometrically optimized via both semiempirical and DFT high theoretical levels. QSAR descriptors were calculated to ascertain the ability of pectin to improve the Oleuropein structure’s hydrophilicity with *Log p* (-1.171). DFT calculations reveal that the proposed Oleu-Pec structures have stable characteristics and high reactivity with dipole moments up to 7.86 Debye. They also proposed that Pec is most likely to interact with Oleu through physical interaction between its OH group and that of Oleu. MESP maps indicated the active sites revealing their tendency to go through either nucleophilic or electrophilic pathways. Furthermore, global reactivity descriptors were calculated to reveal the improved electronic features of Oleu-Pec structures upon adding Pec to Oleu, proposing it for further promising biomedical applications. The integrated approach of docking and molecular dynamics (MD) simulations applied to the P2Y12 receptor in conjunction with the ligands 2-Oleu(OH)-Pec(OH), Oleuropein, Pectin, compared with native inhibitor (AZJ) provides valuable insights into their binding characteristics. Among these ligands, the 2-Oleu(OH)-Pec(OH) emerges as a highly promising candidate, demonstrating exceptional stability with a docking RMSD of 0.68 Å, a robust binding affinity of -6.80 kcal/mol, and an extensive interaction network comprising 18 total bonds, including 12 hydrogen bonds. MD simulations further validate its stability, showcasing a consistent RMSD (~ 0.41 nm), 2–4 intermolecular hydrogen bonds, and the lowest MM/PBSA ΔG value of -54.64 kcal/mol. Principal Component Analysis (PCA) reinforces these findings by revealing the tightest clustering, indicating minimal conformational variability. The synergy observed between Oleuropein and Pectin in the dual-ligand configuration enhances the overall binding strength, surpassing that of the individual components. These outcomes underscore the potential of 2-Oleu(OH)-Pec(OH) as a promising P2Y12 inhibitor for applications in anti-thrombotic therapy.

## Introduction

In the last decades, there has been an increasing trend in scientific research and industry toward using medicinal plants and their metabolites. Their value comes from having little or no adverse effects on the human body, compared to chemically prepared medicines, which could negatively affect the body over time^1^. The *Olea europaea* L. tree significantly enhances the health system in Mediterranean cuisine and culture. Historically, the olive tree’s great attention has come from its religious importance. *The Olea europaea* L. tree belongs to the *Oleaceae* family. Moreover, in ancient times, its leaves, fruits, and oil were used to treat various diseases, including vomiting, fever, malaria, and lower earaches^2,3^.

Oleuropein is the principal phenolic secoiridoid glycoside in all olive tree sections, especially leaves. This biologically active compound recently received more attention due to its pharmacological, nutraceutical, and cosmeceutical value^4,5^. Oleuropein possesses several biological activities, such as antioxidants, anti-inflammatory, cardio- and neuro-protective, anti-cancer, and anti-platelet aggregation^6^. To get the most out of Oleuropein, it must be used in high doses as it has poor absorption in the gut and does not undergo hydrolysis in the stomach as it is resistant to stomach acidity^2^. The disadvantage of high doses of Oleuropein; it may cause gastrointestinal upset, including diarrhea, or abdominal discomfort in sensitive individuals^7,8^. Future studies are needed to establish the relationship between the beneficial effects and the mechanisms of action occurring in the human body in response to the intake of Oleuropein^6^.

Pectin is a biopolymer, *α*-1,4-linked D-galacturonic acid, and *α* -1,2-L-rhamnose unit attached to various neutral sugars. This pharmacophore-active compound has become essential to the research and development of natural medicines and health products with multiple applications because of its structural diversity and complexity^9^. In addition, bioactive pectin and pectin-based composites exhibit improved characteristics to deliver active molecules. Pectin and pectin-based composites serve as interactive matrices by stimulating cell adhesion and cell proliferation and enhancing tissue remodeling by forming an extracellular matrix in vivo.

Several bioactive activities, such as anti-tumor, antibacterial, anti-inflammatory, antioxidant, and immunoregulatory activities, contribute to the pectin’s and pectin-based composite’s enhanced applications in tissue engineering and drug delivery systems^10,11^.

The discovery of new drugs is the main object of pharmacologists and medicinal chemists. It requires their design and synthesis as well as studying their physicochemical, biophysical properties and pharmaceutical functions. The development of drugs has several strategies, and molecular modeling concepts are one of these approaches recently pursued. This approach is often adopted by conducting computational physics calculations to investigate various systems’ physical, chemical, and electronic features for multiple interdisciplinary fields^12^. Moreover, this technique quantifies the relationship between a drug’s physicochemical properties and its resulting biological activity^13^. For instance, quantitative structure-activity relationship parameters (QSAR) are frequently computed for structures with potential applications in biological systems^14^.

Furthermore, molecular electrostatic potential (MESP) maps are utilized to colorfully visualize the charge distribution profiles in chemical systems^15^. MESP maps usually identify the active sites in chemical systems by anticipating the most likely chemical reaction, either nucleophilic or electrophilic^16^. Hence, the computational pharmacology and chemistry of drug-like properties and pharmacokinetic studies have made it more amenable to deciding or predicting a potential drug candidate^17^.

Molecular docking is widely used to predict how small therapeutic compounds interact with their protein targets, providing insights into the affinity and effectiveness of these molecules. This technique is crucial in the rational design of drugs. Given the importance of docking studies in biology and pharmacology, significant efforts have been made to improve algorithms for precise docking predictions^18^. Docking can be employed to assess the interactions between compounds and protein receptors, shedding light on their binding mechanisms and potential biological activities^19–21^.

Recent advancements in computational methodologies have transformed the landscape of drug discovery, enabling precise predictions of ligand-receptor interactions, insights into conformational dynamics, and the systematic identification of off-target effects. Molecular dynamics (MD) simulations, for example, have played a crucial role in elucidating enzymatic activity mechanisms. An excellent illustration is the exploration of the conformational flexibility of the C-site pocket in sirtuin 2, directly influencing inhibitor design^22,23^. Moreover, the integration of hybrid pharmacophore modeling with MD techniques has been successful in enhancing human LTA4H inhibitor optimization by elucidating dynamic binding landscapes. Systems biology strategies, including structure-based network analyses, have further broadened the scope of in-silico tools by associating chymase inhibitors with off-target pathways and opportunities for therapeutic repurposing. During the COVID-19 pandemic, the strength of integrative computational approaches was highlighted as docking, MD simulations, and machine learning cooperatively pinpointed potential inhibitors with significant clinical potential^24,25^.

Targeting the P2Y12 receptor has significant clinical implications, particularly in cardiovascular health, cancer treatment, and neuroprotection. P2Y12 inhibitors, such as clopidogrel and ticagrelor, are crucial for preventing thrombotic events like heart attacks and strokes, often used in combination with aspirin for enhanced protection. Additionally, these receptors play a role in cancer progression by facilitating tumor-platelet interactions, suggesting that P2Y12 inhibitors may also have therapeutic potential in oncology. Furthermore, they may offer neuroprotective benefits in ischemic stroke by reducing inflammation. Based upon what was introduced earlier, our study aims to use the hydrophilic features of pectin structure to improve the biological activity of Oleuropein for further biological applications. Hence, various model molecules are proposed between Oleuropein and pectin, further optimized via semiempirical quantum mechanical calculations and DFT high theoretical level. QSAR descriptors, physical and electronic parameters, and MESP maps are all considered. Moreover, molecular docking and dynamic simulations are utilized to investigate interactions and validate anti-coagulant activity of the hybrid molecule.

## Computational details

###  Quantitative structure activity relationship (QSAR)

The built-up model molecules were geometrically optimized using semi-empirical quantum mechanical calculations at PM6 method via SCIGRESS 3.0 software package at Molecular Modeling and Spectroscopy Laboratory, Centre of Excellence for Advanced Science, National Research Centre, Egypt^26,27^. These optimized structures were utilized to calculate the QSAR parameters and investigate the charge distribution as well as the population of electrons in their orbital in some of the proposed active sites as listed in Table 2.

###  Density function theory calculation (DFT)

For Oleuropien-Pectin proposed structures, computational analyses were performed using Density Functional Theory (DFT). These calculations employed B3LYP/6-311G (d, p) model chemistry^28–30^ using GAUSSIAN 09 software^31^. Highest Occupied Molecular Orbital (HOMO), Lowest Unoccupied Molecular Orbital (LUMO) energies and their related electronic parameters were analyzed. The target compound’s geometry was optimized without constraints, and molecular reactivity indexes were calculated to evaluate drug targeting potential. Several parameters were calculated relying on the energies of both HOMO and LUMO orbitals in each system to quantify its chemical reactivity including ionization potential (I), electronic affinity (A), electronegativity or electronic chemical potential (µ), chemical hardness (η), absolute softness (S), and electrophilicity index (ω), Eqs. (1–6)^32^.

1 $$I = ~ - E_{{HOMO}}$$

2 $$A = ~ - E_{{LUMO}}$$

3 $$\mu = ~\frac{{\left( {E_{{LUMO}} + E_{{HOMO}} } \right)}}{2} = ~\frac{{ - \left( {I + A} \right)}}{2}$$

4 $$\eta = ~\frac{{\left( {E_{{LUMO}} - E_{{HOMO}} } \right)}}{2} = ~\frac{{\left( {I - A} \right)}}{2}$$

5 $$S = \frac{1}{\eta }$$

6 $$\omega = \frac{{\mu ^{2} }}{{2\eta }}$$

###  Molecular docking and dynamics simulations

The human P2Y12 receptor structure (PDB ID: 4NTJ) was chosen based on four key criteria: 1- High Resolution (2.5 Å): This resolution was selected to minimize ambiguity in the binding site. 2- Inclusion of the Co-crystallized Antagonist AZD1283: AZD1283, a clinically validated inhibitor, was present in the structure. This inclusion facilitated a direct comparison with Oleu-Pec binding modes. 3- Human Origin: The receptor structure is of human origin, ensuring relevance for translational research. 3- Structural Completeness: Protonation states were assigned at physiological pH (7.4) using PDB2PQR. In the preprocessing stage, water molecules and non-essential ions were removed, and hydrogen atoms were added using PyMOL. For molecular docking, AutoDock Vina v1.2.3 was utilized. A grid box of dimensions 25 × 25 × 25 Å was centered on AZD binding site (coordinates: x = 15.2, y = − 10.5, z = 5.8). An exhaustiveness value of 32 was set to ensure thorough conformational sampling. The accuracy of the docking protocol was validated by re-docking AZD1283, which resulted in a Root Mean Square Deviation (RMSD) of 1.2 Å compared to its crystallographic pose, confirming the reproducibility of the docking results. The Discovery Studio program was employed to analyze the 2-D interactions between the protein and the ligands. We conducted molecular dynamics (MD) simulations using GROMACS 2022.3 to explore the stability and flexibility of P2Y12 receptor-ligand complexes, employing the CHARMM36 all-atom force field for the protein, the TIP3P water model for solvation, and a topology for the ligand (Oleu-Pec) generated via the CGenFF server with partial charges derived from DFT calculations. The system was solvated in a cubic water box with 1.2 nm padding, neutralized with 0.15 M NaCl, and subjected to energy minimization using the steepest descent algorithm (50,000 steps) to resolve steric clashes, followed by 500 ps NVT equilibration at 300 K with the V-rescale thermostat and 500 ps NPT equilibration at 1 bar with the Parrinello-Rahman barostat. A 100 ns production MD run was performed with a 2-fs time step under periodic boundary conditions, using the LINCS algorithm to constrain hydrogen bonds, the Particle Mesh Ewald method for long-range electrostatics, and a 1.2 nm cutoff for van der Waals interactions. Stability and flexibility were evaluated through root mean square deviation (RMSD) of the protein backbone and ligand, radius of gyration (Rg) for compactness, root mean square fluctuation (RMSF) for residue-specific flexibility, solvent-accessible surface area (SASA), and hydrogen bond occupancy, with trajectories saved every 10 ps and averages computed from the final 100ns (post-equilibration, RMSD plateau < 0.2 nm). Simulations were duplicated for reproducibility^33^.

##  Results and discussion

### Building model molecules

**Fig. 1:**
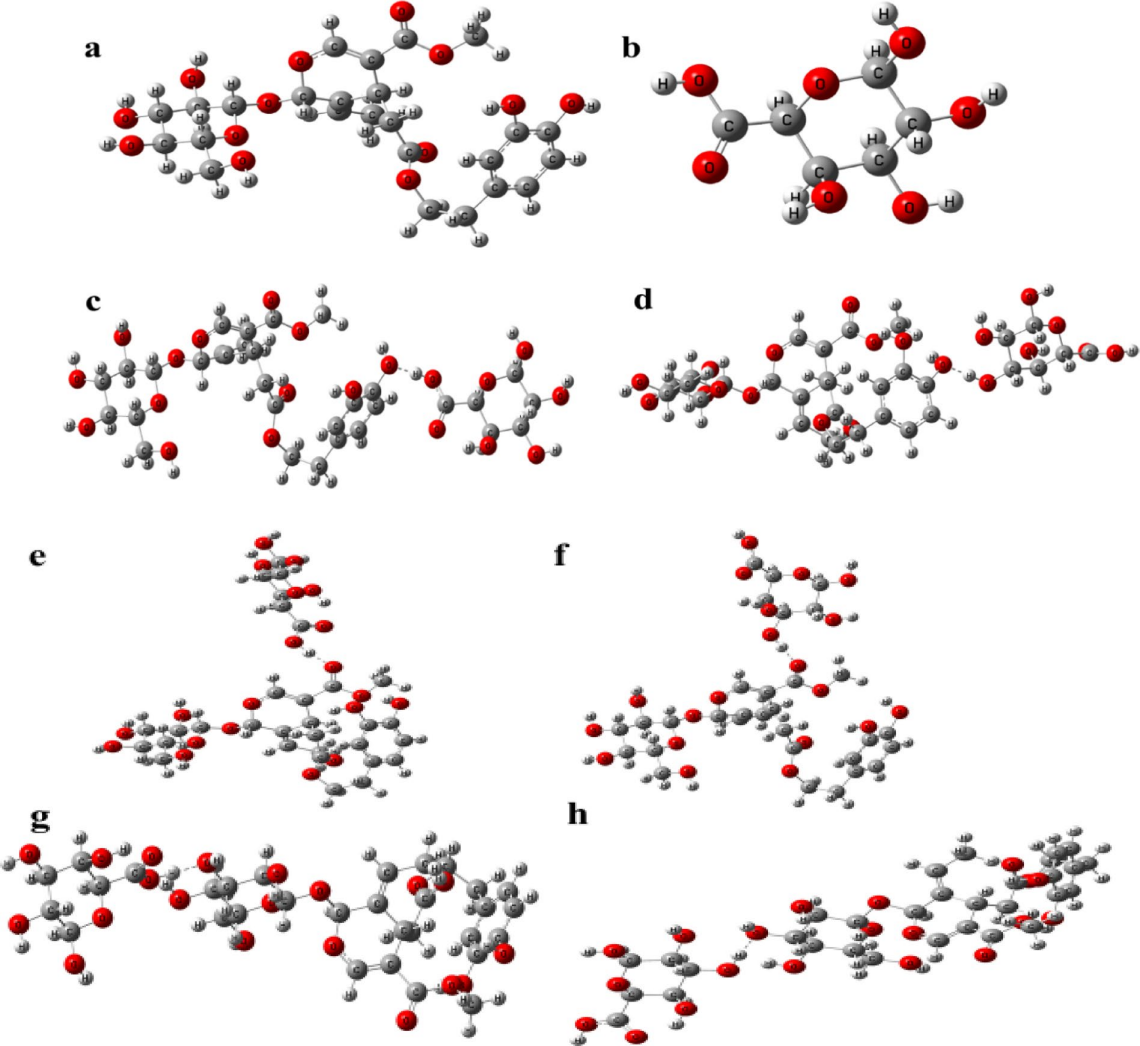
Model molecules of (**a**) oleuropein (Oleu), (**b**) pectin (Pec), (**c**) Oleu (OH)-Pec (COOH), (**d**) Oleu (OH)-Pec (OH), (**e**) Oleu (CO)-Pec (COOH), (**f**) Oleu (CO)-Pec (OH), (**g**) Oleu (OH-Glu)-Pec (COOH) and (**h**) Oleu (OH-Glu)-Pec (OH) calculated at B3LYP/6-311G(d, p) model. [C in grey, H in white and O in red].

Figure 1a depicts the typical Oleuropein (Oleu) molecule structure, presenting a structure rich in functional groups, including several OH groups on both terminals and two CO ones. It is worth noting that Oleu contains a glucose subunit on its terminal which has four OH groups. Hence, we selected three functional groups (two OH and one CO) to be its active sites when interacting with the suggested pectin molecule. On the same manner, pectin structure (Pec.) contains three OH groups and one COOH one linked to an aromatic ring, proposing two active sites for interaction with Oleu structure, Fig. 1b. Consequently, the selected active sites in Oleu and Pec. propose six interaction possibilities. For instance, Fig. 1c and d illustrate the first and second interaction possibilities where the terminals OH group of Oleu interact via physical hydrogen bond with COOH and OH ones of Pec., correspondingly. Similarly, the third and fourth probabilities involve the hydrogen bonding between CO of Oleu and COOH and OH groups of Pec. Figure 1e and f. Finally, the last two interactions occur between one of the OH of glucose subunit (OH-Glu) and COOH and OH ones of Pec., respectively, Fig. 1g and h.

###  Geometry optimization and QSAR descriptors

The proposed model molecules describe the interactions between Oleu. and Pec. molecules were built up and geometrically optimized using PM6 method and some QSAR descriptors were computed and listed in Table 1. Many descriptors can be examined, but this research focused on the most beneficial ones for our aim including total charge, final heat of formation (FF), partition coefficient (Log P), ionization potential (IP), molar refractivity (MR), molecular weight (MWt), polarizability (P), surface area (A) and volume (V).

**Table 1:** Calculated QSAR descriptors of oleu, pec and their proposed interactions including final heat of formation (FF) in kcal/mol, partition coefficient (Log P), ionization potential (IP) in eV, molar refractivity (MR), molecular weight (MWt), polarizability (P) in A^3^, surface area (A) in A^2^ and volume (V) in A^3^.

	FF (kcal/mol)	Log *P*	IP (eV)	MR	MWt (au)	*P* (A^3^)	A (A^2^)	V (A^3^)
Oleu	− 535.735	0.480	− 8.772	127.375	540.514	37.612	493.6	426.22
Pec	− 314.065	− 1.651	− 10.744	35.791	194.139	10.524	163.85	136.5
Oleu (OH)-Pec (COOH) (P_1_)	− 853.222	− 1.171	− 9.091	163.166	734.653	48.441	631.79	556.69
Oleu (OH)-Pec (OH) (P_2_)	− 847.763	− 1.171	− 9.093	163.166	734.653	48.392	650.66	561.13
Oleu (CO)-Pec (COOH) (P_3_)	− 860.188	− 1.171	− 8.848	163.166	734.653	48.239	619.46	551.52
Oleu (CO)-Pec (OH) (P_4_)	− 854.692	− 1.171	− 8.944	163.166	734.653	47.965	639.67	557.27
Oleu (OH-Glu)-Pec (COOH) (P_5_)	− 857.718	− 1.171	− 8.527	163.166	734.653	49.694	616.97	555.15
Oleu (OH-Glu)-Pec (OH) (P_6_)	− 853.669	− 1.171	− 8.774	163.166	734.653	48.950	650.42	556.99

Various QSAR descriptors were calculated as illustrated in Table 1. Since all the proposed structures are in the lowest ground energy state, their charge is zero for neutral structures. The final heat of formation (FF) usually represents the quantity of heat released or acquired from the surroundings to construct a chemical entity^34^. Table 1 depicts that FF is negative for all the proposed models indicating spontaneously constructed chemical structures via exothermic reaction processes. It ranges from − 847.763 to − 860.188 kcal/mol for all the proposed six interactions, indicating that the interaction site has no significant impact on their formation spontaneity.

The partition coefficient or Log P is one of the crucial descriptors in our study where it can determine to what extent the addition of Pec. molecule to Oleu. structure is feasible regarding its application in biological hydrophilic media. To further elaborate, it measures the partitioning of a molecule in both water and octanol solvents. For lipophilic drugs, as the Log Pvalue increases in the positive direction, the lipophilicity also increases (more hydrophobic), which facilitates the movement of the drug across the lipid cell membrane^35^. At the same time, Log P for hydrophilic drugs has a negative value (higher affinity for the aqueous phase)^36^. Hence, the Log P property could also impact the distribution, absorption and pharmacological activity. Table 1 ascertains the hydrophobic nature of Oleu. structure which causes its poor absorption in the gut with a positive value of 0.480, whereas the proposed Pec. structure has a hydrophilic attitude with a negative result of − 1.651.

Moreover, Table 1 supports the proposed idea of adding Pec. to boost the hydrophilic features of Oleu. to facilitate its absorption in the gut. The Log P values of all the suggested interactions have the same negative values of − 1.171, which refers to the same tendency for the aqueous media.

Ionization potential (IP) is the QSAR descriptor that refers to one of the electronic characteristics of a proposed structure where it is often termed as the absorbed amount of energy by the outermost shell electrons to escape from the nuclear forces, and it equals to the HOMO energy of a substance. It is − 8.772 and − 10.744 eV for Oleu. and Pec., correspondingly, ranges from − 8.527 to − 9.093 eV for the proposed Oleu-Pec interactions.

Moreover, Table 1 lists the molar refractivity (MR) and polarizability (P) which represent the tendency of a structure to be polarized in response to an external field. Both rely on the molar volume of the chemical system according to the Lorentz-Lorenz equation which correlates the MR with the structure’s volume and polarizability^37,38^, Eq. (7).

7 $$MR = \frac{{n_{D}^{2} - 1}}{{n_{D}^{2} + 2}}V_{m} = \frac{{4\pi }}{3}N_{A} \alpha$$

where, n is the substance’s refractive index, V_m_ is its molar volume, N_A_ is Avogadro’s number and polarizability. Consequently, Oleu has larger MR and P values consistent with the Lorentz–Lorentz equation. Since the proposed interactions depend on the same two individuals (Oleu and Pec), their MR and P values have a narrow range. For instance, P ranges from 47.965 to 49.694 A3, whereas all have the same MR value of 163.166.

Moreover, Table 2 lists the charge distribution, number of electrons and their population in the s and p orbitals of some of the elements composing some active sites.

**Table 2:** Net atomic charge distribution and electronic configurations of candidates proposed structures calculated at PM6 method.

Atom	Structure	Charge	No. of electrons	s-Pop	*p*-Pop
O3	Oleu	− 0.5510	6.5510	1.8236	4.7274
	Oleu− Pec (P_5_)	− 0.5563	6.5563	1.8274	4.7289
	Oleu-Pec (P_6_)	− 0.5609	6.5609	1.8269	4.7340
O11	Oleu	− 0.5455	6.5455	1.8531	4.6924
	Oleu-Pec (P_3_)	− 0.6096	6.6096	1.8496	4.7600
	Oleu-Pec (P_4_)	− 0.6045	6.6045	1.8528	4.7517
O13	Oleu	− 0.4475	6.4475	1.8102	4.6373
	Oleu-Pec (P_1_)	− 0.4718	6.4718	1.8079	4.6639
	Oleu-Pec (P_2_)	− 0.4876	6.4876	1.8075	4.6801
H21	Oleu	0.3242	0.6758	0.6758	
	Oleu-Pec (P_2_)	0.3466	0.6534	0.6534	
	Oleu-Pec (P_4_)	0.3412	0.6588	0.6588	
	Oleu-Pec (P_6_)	0.3589	0.6411	0.6411	
H23	Oleu	0.3486	0.6514	0.6514	
	Oleu-Pec (P_1_)	0.3992	0.6008	0.6008	
	Oleu-Pec (P_3_)	0.3998	0.6002	0.6002	
	Oleu-Pec (P_5_)	0.4086	0.5914	0.5914	

Table 2 lists the distribution of charges, number of electrons and their population in some active sites of the molecular orbitals of Oleu, Pec and Oleu-Pec interactions. It illustrates the impact of interacting Pec with Oleu on the number of electrons and their population in s and p orbitals. For instance, the O_11_ atom has a negative charge of − 0.5455 in Oleu which increases to -0.6096 and − 0.6045 in Oleu-Pec (P_3_) and Oleu-Pec (P_4_), respectively. This rise in negative charge comes on account of its neighbouring less electronegative H atom. Furthermore, H_23_ atom has a positive charge of + 0.3486 in Oleu which increases to + 0.39922, + 0.39978 and + 0.4086 in Oleu-Pec (P_1_), Oleu-Pec (P_3_) and Oleu-Pec (P_5_), respectively. This clearly refers to the impact of interacting Pec with Oleu structure on redistributing the electron populations in the resulting structures.

###  DFT geometry optimization and MESP maps

In addition to optimizing the structures of Oleu and Pec via semiempirical PM6 method, they were reoptimized at higher DFT level using B3LYP/6-311G (d, p) theoretical model. Table 3 presents some of the extracted physical and electronic features after structures’ optimization including energy (E), dipole moment (TDM) and HOMO/LUMO bandgap energy (ΔE).

**Table 3:** Calculated total energy (E) as kev, total dipole moment (TDM) as Debye and HOMO/LUMO bandgap energy (ΔE) as eV of oleuropein, pectin and their proposed interactions at B3LYP/6-311G(d, p) theoretical model.

	E (keV)	TDM (Debye)	ΔE (eV)
Oleu	− 53.0708	3.8591	5.1852
Pec	− 20.7195	4.2686	6.4467
Oleu (OH)-Pec (COOH) (P_1_)	− 73.7907	4.6758	5.5011
Oleu (OH)-Pec (OH) (P_2_)	− 73.7916	6.5707	4.5737
Oleu (CO)-Pec (COOH) (P_3_)	− 73.7918	5.7909	4.1226
Oleu (CO)-Pec (OH) (P_4_)	− 73.7908	1.2478	5.0804
Oleu (OH-Glu)-Pec (COOH) (P_5_)	− 73.7910	5.8862	5.1408
Oleu (OH-Glu)-Pec (OH) (P_6_)	− 73.7910	7.8616	5.0156

Table 3 lists some extracted physical parameters frequently regarded as indicators of the structure’s characteristics. For instance, total energy is always considered the main physical descriptor of chemical stability. The Oleu and Pec structures calculated total energies equal − 53.0708 and − 20.7195 keV, respectively. The interaction between Pec and Oleu produces structures with significantly lower energy, signifying highly stable entities. For example, the total energies of all the proposed Oleu-Pec structures have nearly the exact value of -73.79 keV. Wherever the interaction site is, this could validate Oleu’s and Pec’s capacity to construct stable structures.

Moreover, TDM is another electronic feature that refers to the reactivity of chemical substances^39^. It determines to what extent an atom can interact with another neighboring one due to its dominant charges. The calculated TDM of both Oleu and Pec structures equal 3.8591 and 4.2686 Debye, correspondingly. Adding Pec to Oleu produces chemical structures with larger TDM and hence greater chemical reactivity toward their neighbors, except the fourth interaction probability occurs between the CO group of Oleu and OH one of Pec with only 1.2478 Debye. However, the other five interactions have larger TDM values ranging from 4.6758 for P1 to 7.8616 Debye for P_6_.

Furthermore, bandgap energies are often considered to represent the energy acquired by the valence electron to move toward the conduction band away from the nuclear attraction force. Oleu and Pec structures have bandgap energies of 5.1852 and 6.4467 eV, correspondingly. However, the interaction of Pec with Oleu has little impact on the resulting bandgaps of most of the proposed interactions. For instance, only the P_2_ and P_3_ have lower bandgap relative to all the other structures with a bandgap of 4.5737 and 4.1226 eV, respectively. The other four possibilities have bandgap energies similar to the Oleu structure around 5.1 eV. The DFT calculations conducted recommend that both P_2_ and P_6_ interactions occur between Oleu and Pec, since they have the largest TDM and quite low bandgap energies. It is worth noting that both P_2_ and P_6_ interactions occur between the OH of Pec and that of Oleu either in the glucose moiety or on the other side of the Oleu structure.

### Molecular electrostatic potential (MESP) maps

**Fig. 2:**
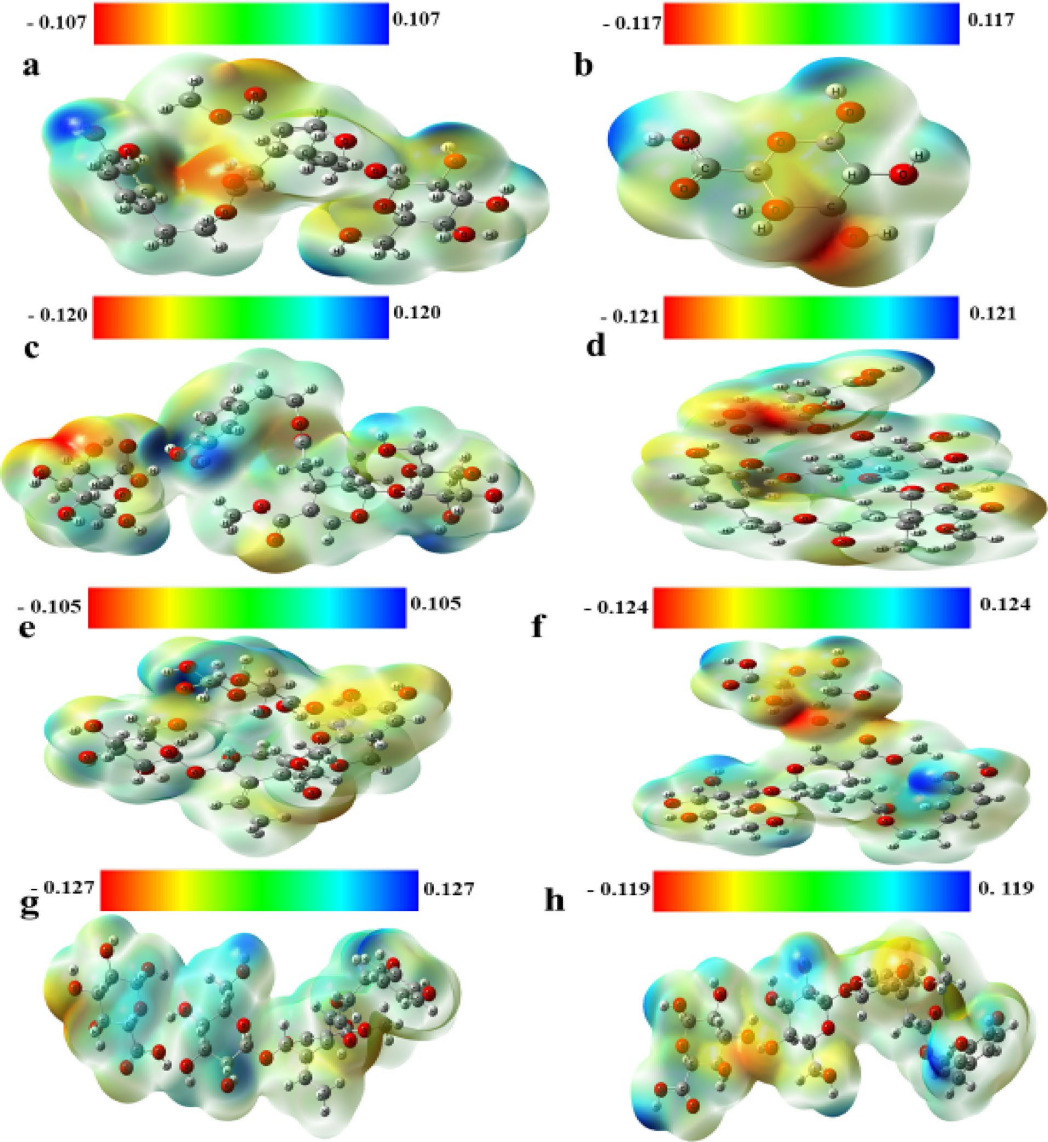
MESP maps of (**a**) oleuropein (Oleu), (**b**) pectin (Pec), (**c**) Oleu (OH)-Pec (COOH), (**d**) Oleu (OH)-Pec (OH), (**e**) Oleu (CO)-Pec (COOH), (**f**) Oleu (CO)-Pec (OH), (**g**) Oleu (OH-Glu)-Pec (COOH) and (**h**) Oleu (OH-Glu)-Pec (OH)calculated at B3LYP/6-311G (d, p) model.

MESP maps were constructed for the geometrically optimized structures of Oleu, Pec and their proposed interactions at the same DFT/B3LYP/6-311G (d, p) theory model, as depicted in Fig. 2. Since MESP maps could present valuable data about the electron density distribution and together with the hydrogen bond interactions through chemical structures^40^, they are frequently utilized to investigate the potential active sites determining the most probable pathway that a structure would adopt. They could determine whether a specific active site would likely go through a nucleophilic or an electrophilic interaction. Further, if the active site has high electron density (appears in red), it would prefer to be involved in a nucleophilic interaction and vice versa^41^. In other words, MESP maps appear in a range of rainbow colors from red to orange, yellow, green to light and dark blue to represent the gradient of the most negative, the neutral to the most positive regions, respectively.

In general, all the constructed MESP maps are characterized by having a unique color distribution referring to good charge distribution nature. This may be related to the presence of several atoms of a broad difference in electronegativity, such as H, C, N and O. These configurations can be correlated with the presence of the tendency of the proposed structures to go through both nucleophilic and electrophilic pathways. For instance, the MESP map of Oleu appears in greenish around the aromatic rings of Oleu structure with red regions -CO groups and blue near the edges where the terminal H atoms exist. On contrary to Oleu, the MESP map of Pec comprises mainly of red regions and blue ones for high and low electron density sites, respectively. The interaction of Pec with Oleu structure boosts the charge distribution profiles in the resulting structures. Most of the proposed structures are characterized by red color around the interaction site referring to a region of high electron density due to the conducted interaction. However, the P_5_ interaction appears mostly in light blue color indicating a slightly positively charged structure preferring electrophilic pathways with neighbors.

### Global reactivity descriptors

Several parameters were calculated relying on the energies of both HOMO and LUMO orbitals in the optimized systems at B3LYP/6-311G (d, p) model to quantify their chemical reactivity including ionization potential (I), electronic affinity (A), electronegativity or electronic chemical potential (µ), chemical hardness (η), absolute softness (S), and electrophilicity index (ω), as listed in Table 4.

**Table 4:** Calculated frontiers parameters for oleu, pec and their proposed interactions at B3LYP/6-311G(d, p) model including ionization potential (I), electronic affinity (A), electronic chemical potential (µ), chemical hardness (η), absolute softness (S) and electrophilicity index (ω).

	I (eV)	A (eV)	µ (eV)	η (eV)	S (eV)^−1^	ω (eV)
Oleu	5.8856	0.7004	− 3.2930	2.5926	0.3857	2.0913
Pec	7.2353	0.7886	− 4.0119	3.2234	0.31024	2.4967
Oleu (OH)-Pec (COOH) (P_1_)	6.3030	0.8019	− 3.5525	2.7505	0.3636	2.2941
Oleu (OH)-Pec (OH) (P_2_)	5.5743	1.0006	− 3.2874	2.2869	0.4373	2.3629
Oleu (CO)-Pec (COOH) (P_3_)	5.4723	1.3497	− 3.4111	2.0613	0.4851	2.8222
Oleu (CO)-Pec (OH) (P_4_)	5.9642	0.8838	− 3.4240	2.5402	0.3937	2.3077
Oleu (OH-Glu)-Pec (COOH) (P_5_)	6.0301	0.8893	− 3.4596	2.5704	0.3890	2.3283
Oleu (OH-Glu)-Pec (OH) (P_6_)	5.8981	0.8825	− 3.3903	2.5078	0.3988	2.2916

Table 4 lists some of the calculated global reactivity descriptors that would represent structures’ reactivity in a quantitative manner. Ionization potential is the additive inverse of the energy of HOMO orbital; hence it represents the energy acquired to eject an electron from the valence band overcoming its binding to the nucleus. The computed ionization potential of Oleu and Pec equals 5.88.56 and 7.2353 eV, respectively. However, the addition of Pec to Oleu had little effect on the resulting ionization potential of Oleu-Pec structures. For instance, Oleu-Pec structures have an ionization potential range from 5.4723 for P_3_ to 6.3030 for P_1_. On the same manner, electronic affinity (A) is the additive inverse of the energy of LUMO energy referring to the acquired energy by a chemical system to accept electrons into its LUMO orbital. Table 4 depicts that electronic affinity has the same attitude as ionization potential where Pe structure has larger electronic affinity energy relative to that of Oleu with 0.7886 and 0.7004 eV, respectively. Likewise, the interaction of Pec with Oleu results in a slight rise in the Oleu-Pec structures where their electronic affinity ranges from 0.8019 for P_1_ to 1.3496 eV for P_3_ agreeing with the results of ionization potential to a great extent. Then, electronegativity or the electronic chemical potential (µ) is the ability of a chemical system to attract electrons from neighbors for longer intervals that is charges often move from the structure of lower electronegativity to that of the higher one when interacting with each other according to HSAB theory^42^. Hence, it depends on the mean value of both E_LUMO_ and E_HOMO_. Oleu and Pec have electronegativity values of -3.2930 and − 4.0119 eV, correspondingly, which fluctuates around − 3.4 eV for Oleu-Pec structures ranging from − 3.5525 for P_1_ to − 3.2874 for P_2_ referring to similar ability to attract electrons from neighbors in the surrounding area. Moreover, the electronic softness represents the ease in which a structure donates its charges. The S results reveal that P_1_ (0.3636 eV) has the less tendency to donate its electrons whereas the P_3_ (0.4851 eV) has the largest one coinciding with the calculated electronegativity results.

### Molecular docking of P2Y12 protein receptor

**Fig. 3:**
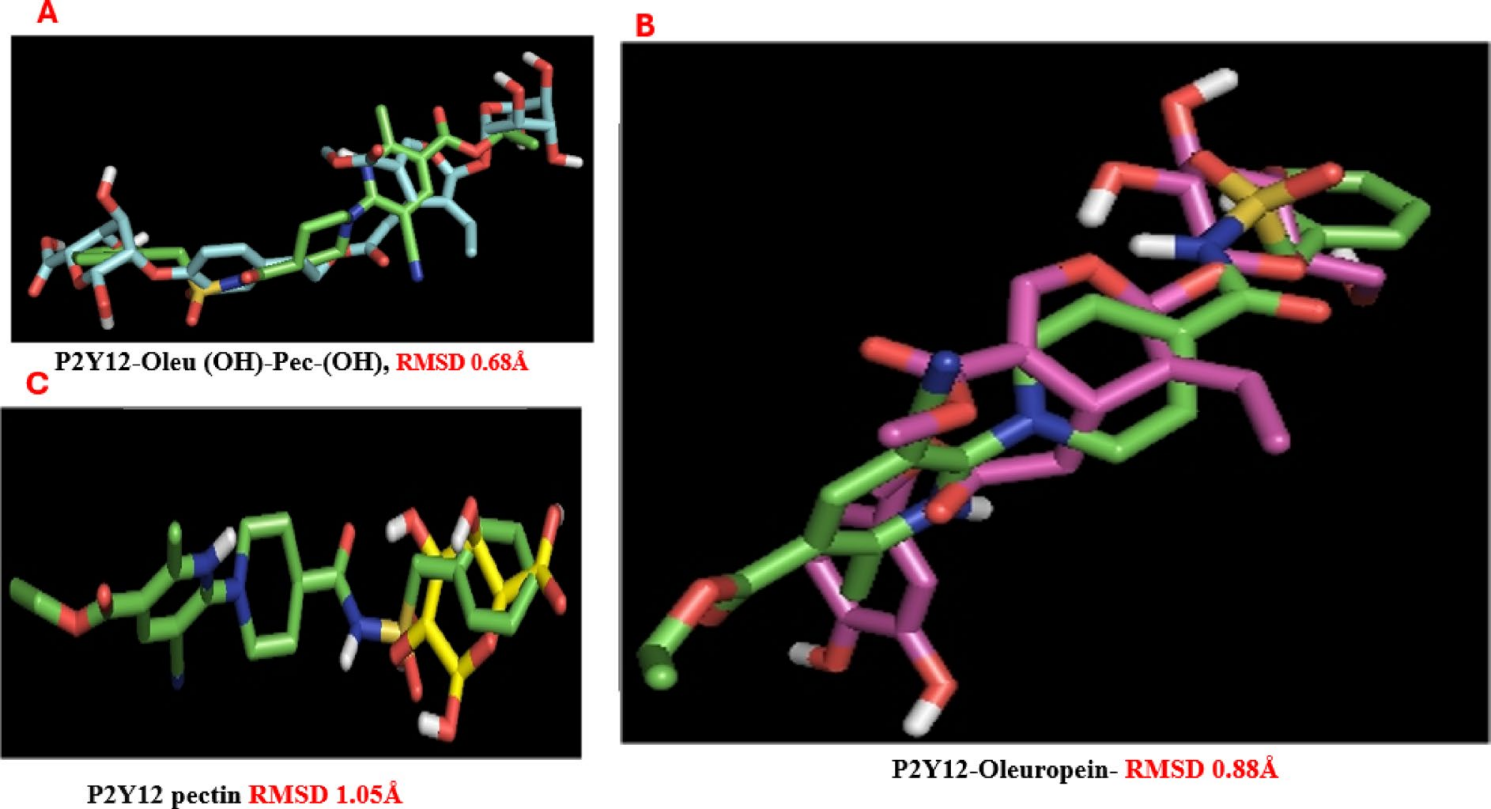
RMSD values for P2Y12 receptor complexes: (**A**) oleuropein-pectin dual-ligand (0.68 Å), (**B**) pectin alone (1.05 Å), and (**C**) oleuropein alone (0.88 Å).

The docking analysis results for the binding energies of the compound molecules are presented in Table 5 and Fig. 3. The molecular docking study of ligands with the P2Y12 receptor (PDB: 4PXZ) reveals significant differences in binding stability, as measured by RMSD values. The dual-ligand complex, 2-Oleu(OH)-Pec(OH), exhibits a low RMSD of 0.68 Å, indicating high structural stability and a close fit to the reference structure, nearly matching the native ligand AZJ (0.65 Å). Oleuropein alone has a moderate RMSD of 0.88 Å, suggesting reasonable stability, while Pectin’s higher RMSD of 1.05 Å reflects poorer binding stability. Notably, the compound (Oleu (OH)-Pec-(OH)) formed twelve hydrogen bonds with key amino acids such as Tyr105, Ser101, Gln263, Lys80, Asp84, Arg256, Lys173, Val102, Phe24, and Cys194. Additionally, various hydrophobic interactions were identified within the active site, including Carbon-H bonds with Phe106 and Ser156, and Pi-Alkyl interactions with Val102, Phe277, Leu284, and Tyr105. Oleuropein, on the other hand, established four hydrogen bonds with critical amino acids, including Cys194, Arg256, Tyr259, and Tyr105, alongside several hydrophobic interactions such as Carbon-H bonds with Ser156 and His187, Pi-Alkyl interactions with Cys175 and Leu284, Pi-Cation interactions with Lys80, Pi-Sulfur interactions with Cys175, and Pi-Pi stacking with Phe104. Lastly, pectin formed five hydrogen bonds with essential amino acids like Val102, Phe106, Met152, and Cys194, along with two Carbon-H bonds with Phe106 and Tyr105. Importantly, the amino acids Tyr259, Lys80, and Cys194 in the catalytic site were conserved across interactions with both the compounds and the native ligands, enhancing the binding affinity. Collectively, these findings suggest that (Oleu (OH)-Pec-(OH)), are likely to effectively target the P2Y12 receptor (Table 6). Also, these results suggest that the combination of Oleuropein and Pectin in the dual-ligand enhances conformational stability, likely due to synergistic interactions with the receptor’s binding pocket. Additionally, binding affinities calculated using PyRx, AutoDock Vina, rDock, and SwissDock highlight the dual-ligand’s superior performance. The 2-Oleu(OH)-Pec(OH) complex achieves affinities ranging from − 6.65 to − 6.90 kcal/mol (average ~ − 6.76 kcal/mol), surpassing Oleuropein (− 6.20 to − 6.45 kcal/mol, average ~ − 6.34 kcal/mol) and Pectin (− 4.70 to − 5.01 kcal/mol, average ~ − 4.82 kcal/mol). The native ligand AZJ shows slightly better affinities (− 6.50 to − 6.72 kcal/mol, average ~ − 6.65 kcal/mol).

**Fig. 4:**
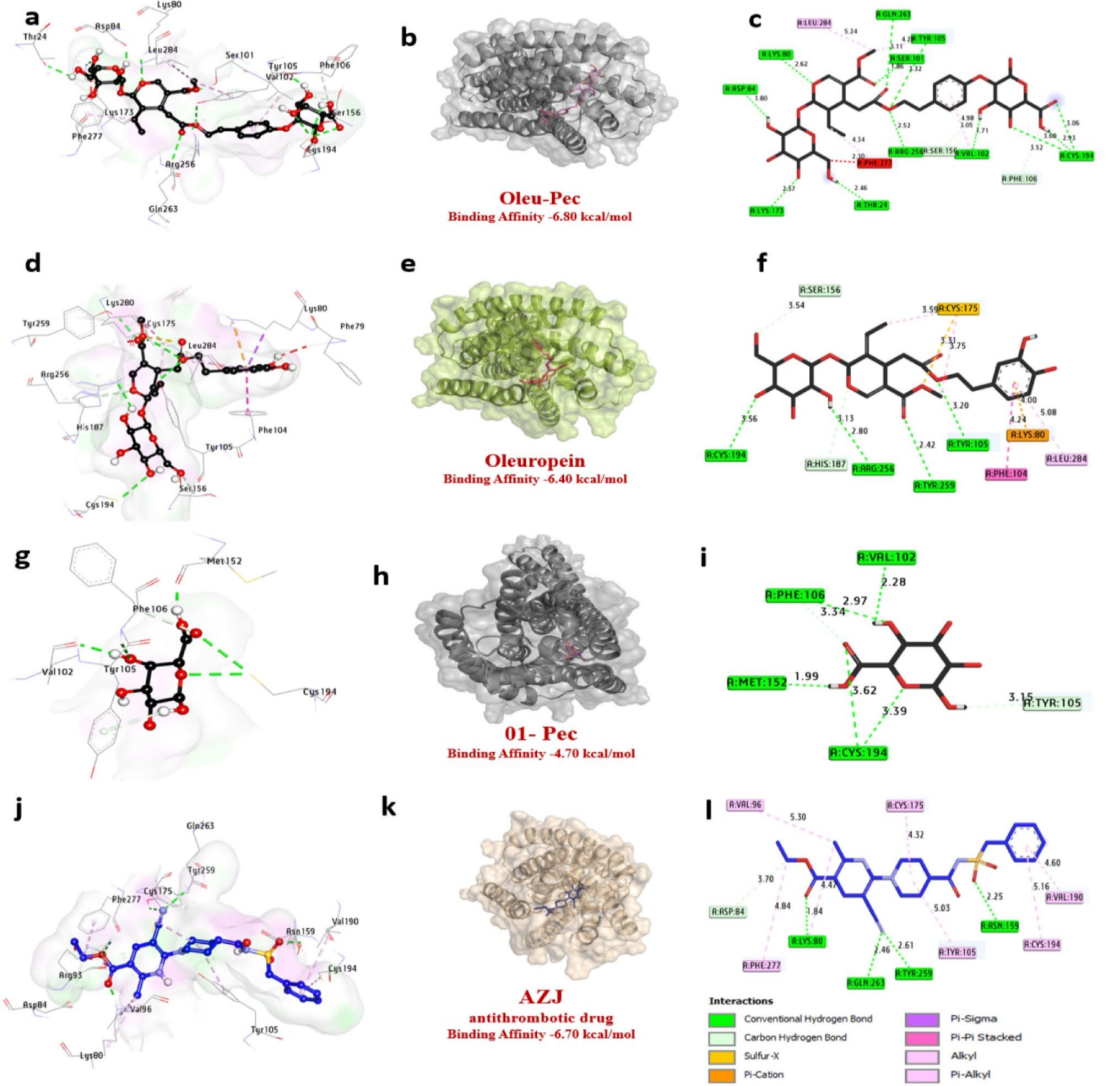
3D representations of a compound at the binding pocket of the crystal structure of P2Y_12_ receptor protein (PDB: ID 4PXZ): (**a**,** b**, and** c**) P_2_ (Oleu (OH)-Pec-(OH)), (**d**, **e**, and **f**) oleuropein, (**g**, **h** and **i**) pectin, (**j**, **k** and **l**) AZJ (native ligand).

In Fig. 4; The dual-ligand’s strong affinity, driven by interactions with Lys80, Tyr105, and Arg256, positions it as a promising P2Y12 inhibitor. Moreover, the dual-ligand forms an extensive interaction network with the P2Y12 receptor, including 12 hydrogen bonds (H-bonds) with residues such as Tyr105, Lys80, and Cys194 (bond lengths: 1.71–3.68 Å) and 6 hydrophobic contacts (e.g., Pi-Alkyl with Val102). With 18 total bonds, it outperforms Oleuropein (4 H-bonds, 12 total bonds), Pectin (5 H-bonds, 7 total bonds), and AZJ (4 H-bonds, 12 total bonds). Pectin’s limited interactions, primarily with Cys194 and Val102, explain its weak affinity (− 4.70 kcal/mol). The dual-ligand’s high H-bond count and diverse hydrophobic contacts correlate with its low RMSD (0.68 Å) and strong affinity (-6.80 kcal/mol), suggesting a robust binding mechanism.

The consistency across docking tools, which employ gradient-based, stochastic, and hybrid algorithms, underscores the reliability of these findings. The dominance was consistent across all algorithms, with AutoDock predicting the strongest binding due to robust conformational sampling, while rDock (stochastic search) and SwissDock (gradient-based/hybrid approaches) yielded intermediate values. Key residues like Cys194 emerged as critical for hydrophobic anchoring, aligning with prior P2Y12 studies emphasizing Lys80/Tyr105’s role in binding antagonists. These findings mirror GPCR dual-ligand research, such as adenosine A2A receptor studies where synergistic H-bond/π-alkyl networks similarly enhanced stability (RMSD < 1.0 Å). While scoring functions may overemphasize H-bonds (explaining Pectin’s weaker performance), consensus across tools validated the dual-ligand’s therapeutic potential. Overall, the computational synergy between Oleu-Pec and its alignment with established GPCR mechanisms positions as a promising candidate for P2Y12 inhibition, warranting experimental exploration to bridge these insights with clinical application. These results were similar to Xiao et al.,^43^ employed a combination of functional analysis, network pharmacology, molecular docking, and animal experiments to explore the P2Y12 receptor-independent antiplatelet targets and the biological mechanisms by which cryptotanshinone improves thrombosis. Also, Halim et al.^44^ who obtained that P2Y12 is considered as a potent target to inhibit platelet aggregation in thrombotic and cardiac emergencies. This research focuses on in silico inhibition of P2Y12 by structure-based drug design techniques.

**Table 5:** Binding affinity of different docking tools with different algorithms, key amino acids and RMSD values.

NO	Ligands	Pyrx	Autodock Vina	rDock	SwissDock	Key amino acids	RMSD values Å
		Gradient-based optimization	Gradient-based optimization	Stochastic search (genetic algorithm + simulated annealing)	Hybrid approach (evolutionary + systematic search)		
1	2- Oleu (OH)-Pec (OH)	− 6.65	− 6.80	− 6.70	− 6.70	Lys80 Tyr105 Arg256	0.680
2	Oleuropein	− 6.20	− 6.40	− 6.35	− 6.30	Lys80 Tyr105 Arg256	0.880
3	Pectin	− 4.75	− 4.70	− 4.60	− 4.80	Lys80 Tyr105 Cys194	1.050
4	AZJ	− 6.50	− 6.70	− 6.60	− 6.66	Lys80 Tyr105 Arg256	0.650

**Table 6:** Molecular interactions with amino acids of the crystal structure of P2Y12 receptor (PDB: ID 4PXZ).

No	Protein	Ligands	3D structure	Hydrophilic interactions	Hydrophobic contacts			No. of H-bonds	No. of total bonds	Affinity kcal mol^− 1^
				Residue (H- bond)	Length	Residue (Bond type)	Length			
1	Crystal structure of P2Y12 receptor (PDB: ID 4PXZ)	2- Oleu (OH)-Pec (OH)		Tyr105, (H- Bond) Ser101, (H- Bond) Gln263, (H- Bond) Lys80, (H- Bond) Asp84, (H- Bond) Arg256, (H- Bond) Lys173, (H- Bond) Val102, (H- Bond) Phe24, (H- Bond) Cys194, (H- Bond) Cys194, (H- Bond) Cys194, (H- Bond)	3.32 1.86 3.11 2.62 1.80 2.52 2.57 1.71 2.46 3.68 2.93 3.06	Phe106, (Carbon-H bond) Ser156, (Carbon-H bond) Val102, (Pi-Alkyl) Phe277, (Pi-Alkyl) Leu284, (Pi-Alkyl) Tyr105, (Pi-Alkyl)	3.52 3.05 4.98 4.34 5.24 4.24	12	18	− 6.80
2		Oleuropein		Cys194, (H- Bond) Arg256, (H- Bond) Tyr259, (H- Bond) Tyr105, (H- Bond)	3.56 2.80 4.42 3.20	Ser156, (Carbon-H bond) His187, (Carbon-H bond) Cys175, (Pi-Alkyl) Cys175, (Pi-Alkyl) Leu284, (Pi-Alkyl) Lys80, (Pi-Cation) Cys175, (Pi-Sulfur) Phe104, (Pi-Pi Stacked)	3.54 3.13 3.59 3.75 5.08 4.00 3.31 4.24	4	12	− 6.40
3		Pectin		Val102, (H- Bond) Phe106, (H- Bond) Met152, (H- Bond) Cys194, (H- Bond) Cys194, (H- Bond)	2.28 2.97 1.99 3.62 3.39	Phe106, (Carbon-H bond) Tyr105, (Carbon-H bond)	3.34 3.15	5	7	− 4.70
4		AZJ (Native ligand)		Tyr259, (H- Bond) Lys80, (H- Bond) Asn159, (H- Bond) Gln263, (H- Bond)	2.56 1.84 2.25 2.61	Asp84, (Carbon-H bond) Val190, (Pi-Alkyl) Cys194, (Pi-Alkyl) Tyr105, (Pi-Alkyl) Cys175, (Pi-Alkyl) Val96, (Pi-Alkyl) Phe277, (Pi-Alkyl) Lys80, (Pi-Alkyl)	3.70 4.60 5.16 5.03 4.32 5.30 4.84 4.47	4	12	− 6.70

### Dynamic simulations of P2Y12 receptor protein

**Fig. 5:**
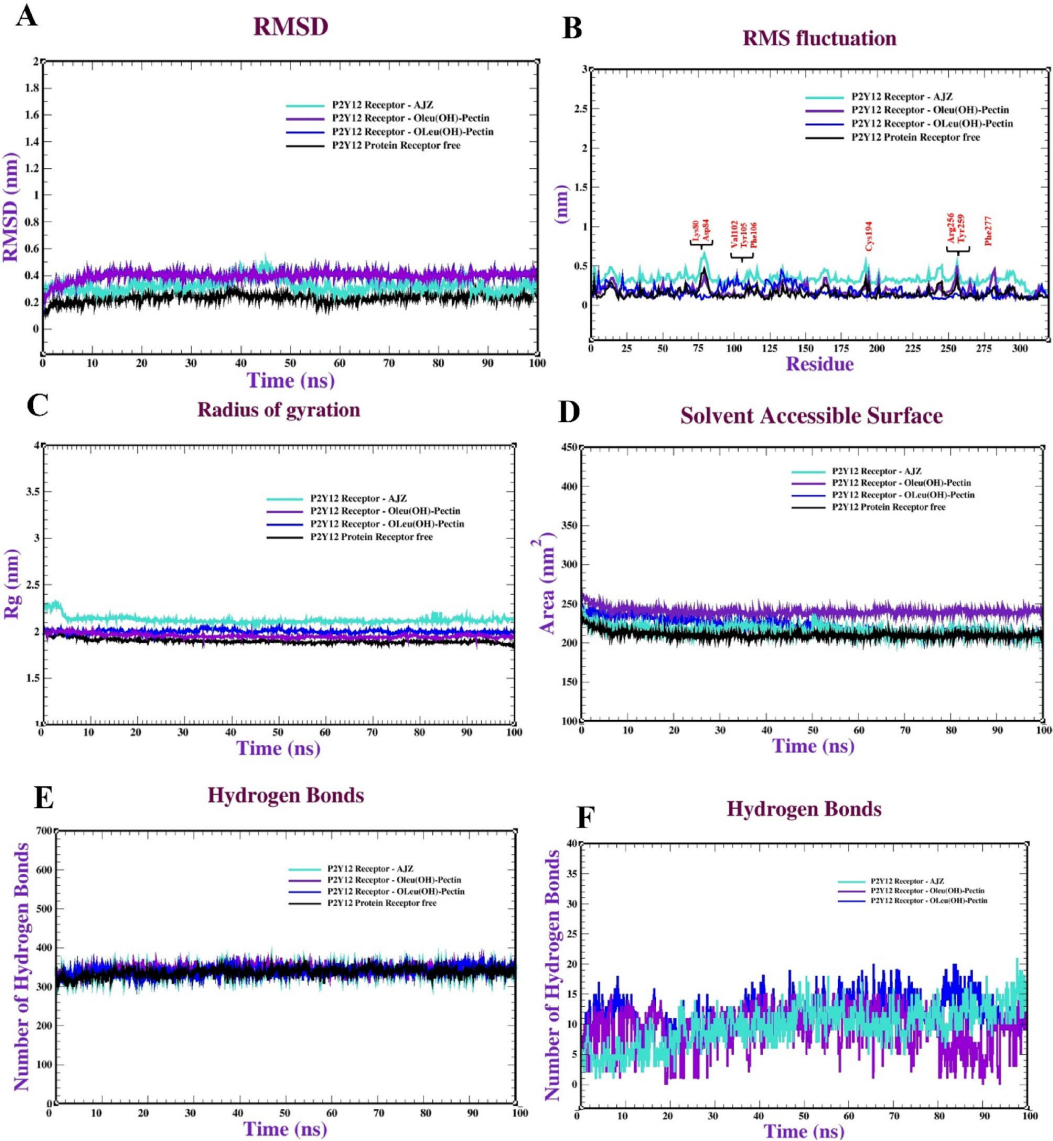
Molecular dynamics of the P2Y12 receptor (PDB: ID 4PXZ): complexed with P_2_ (Oleu (OH)-Pec-(OH)) : (**a**) RMSD, (**b**) RMSF, (**c**) radius of gyration (R_g_), (**d**) SASA, (**e**) Intramolecular hydrogen bonds and (**f**) Intermolecular hydrogen bonds.

**Fig. 6:**
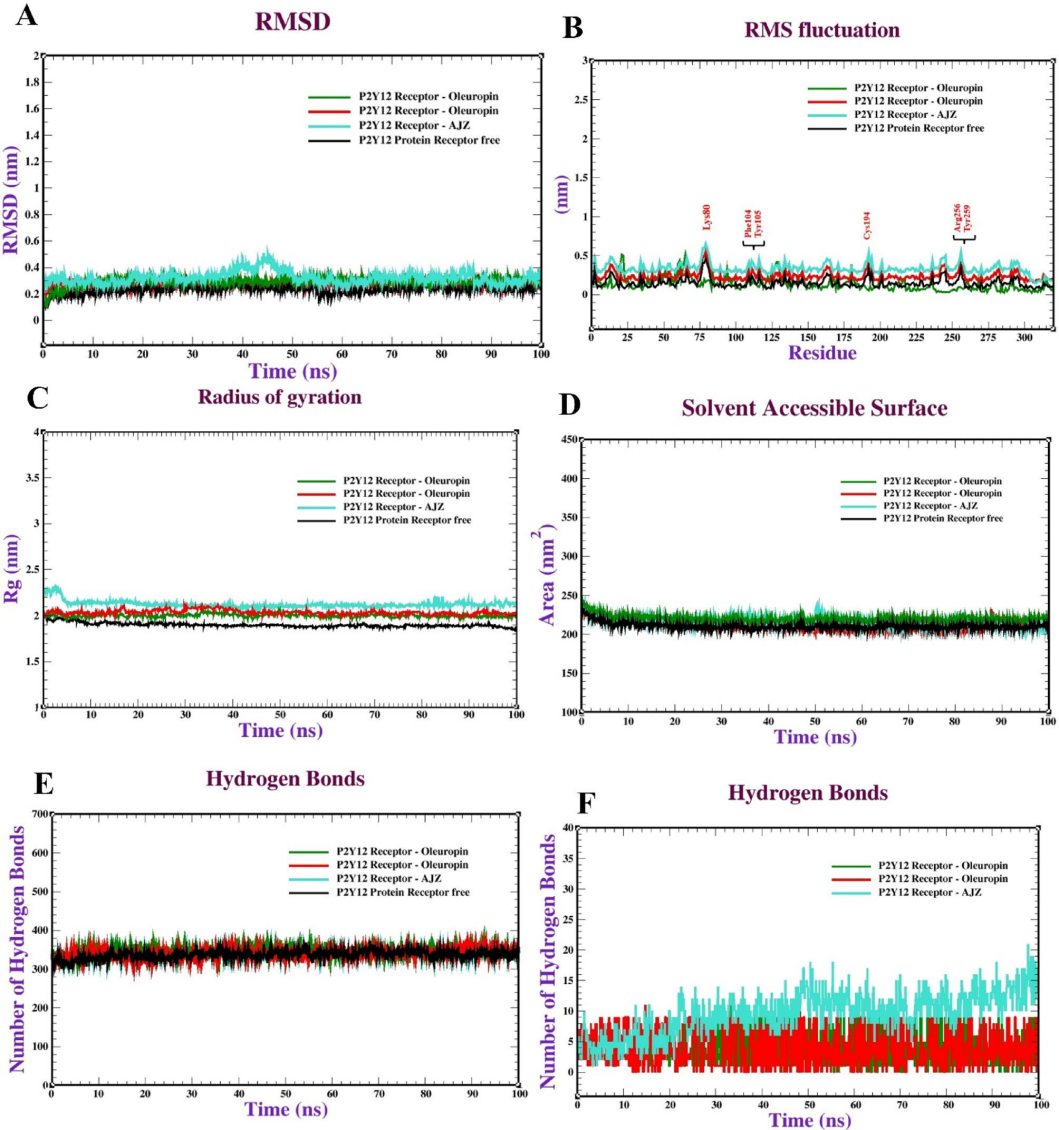
Molecular dynamics of the P2Y12 receptor (PDB: ID 4PXZ): complexed with oleuropein: (**a**) RMSD, (**b**) RMSF, (**c**) radius of gyration (R_g_), (**d**) SASA, (**e**) intramolecular hydrogen bonds and (**f**) intermolecular hydrogen bonds.

Molecular dynamics (MD) simulations offer significant insights into molecular behaviour over time, enabling the observation of conformational changes that may arise during interaction. Post-docking, these simulations can compute binding free energy and interaction energies with enhanced precision, which is essential for assessing the stability of expected complexes and determining the most advantageous binding modes. Ultimately, MD simulations can corroborate or contest docking predictions by illustrating whether the expected complexes maintain stability under physiological settings. Molecular dynamics simulations of the P2Y12 protein complexed with 2-Oleu (OH)-Pec (OH), oleuropein, and pectin involved multiple studies to evaluate the stability and dynamics of the protein complexes. The molecular dynamics simulation of the P2Y12 receptor complexed with 2-Oleu(OH)-Pec(OH) (Fig. 5) demonstrates exceptional stability and robust interactions over 100 ns. The RMSD plot (panel a) stabilizes at ~ 0.4 nm (4 Å) after 10 ns with minimal fluctuations (~ 0.01 nm), indicating a highly stable binding conformation, consistent with the docking RMSD of 0.68 Å. The RMSF (panel b) shows low residue fluctuations (< 0.2 nm) except in flexible loop regions (~ 0.3–0.4 nm), suggesting a rigid binding site. SASA (panel c) remains steady at 150–160 nm², and Rg (panel d) stabilizes at ~ 2.4 nm, reflecting consistent solvent exposure and compactness. The complex maintains ~ 300–350 intramolecular H-bonds (panel e) and forms 2–4 intermolecular H-bonds with occasional peaks to 5 (panel f), aligning with the docking’s 12 H-bonds and 18 total bonds. These results highlight the dual-ligand’s strong and stable binding, driven by synergistic interactions, making it a promising P2Y12 inhibitor candidate. Figure 6 illustrates the MD behavior of the P2Y12-Oleuropein complex, revealing moderate stability. The RMSD (panel a) rises to ~ 0.3 nm (3 Å) by 100 ns with fluctuations (~ 0.05 nm), suggesting less stability than the dual-ligand (docking RMSD: 0.88 Å). RMSF (panel b) is similar to the dual-ligand (< 0.2 nm, peaks at ~ 0.3 nm in loops), indicating comparable receptor flexibility. SASA (panel c) fluctuates between 140 and 150 nm², and Rg (panel d) stabilizes at ~ 2.45 nm, indicating slight conformational variability and reduced compactness. Intramolecular H-bonds (panel e) remain stable (~ 300–340), but intermolecular H-bonds (panel f) range from 1 to 3, occasionally dropping to 0, reflecting weaker ligand-receptor interactions compared to the dual-ligand’s 2–4 H-bonds. These findings align with docking’s 4 H-bonds and lower affinity (-6.40 kcal/mol), confirming Oleuropein’s moderate binding strength.

**Fig. 7:**
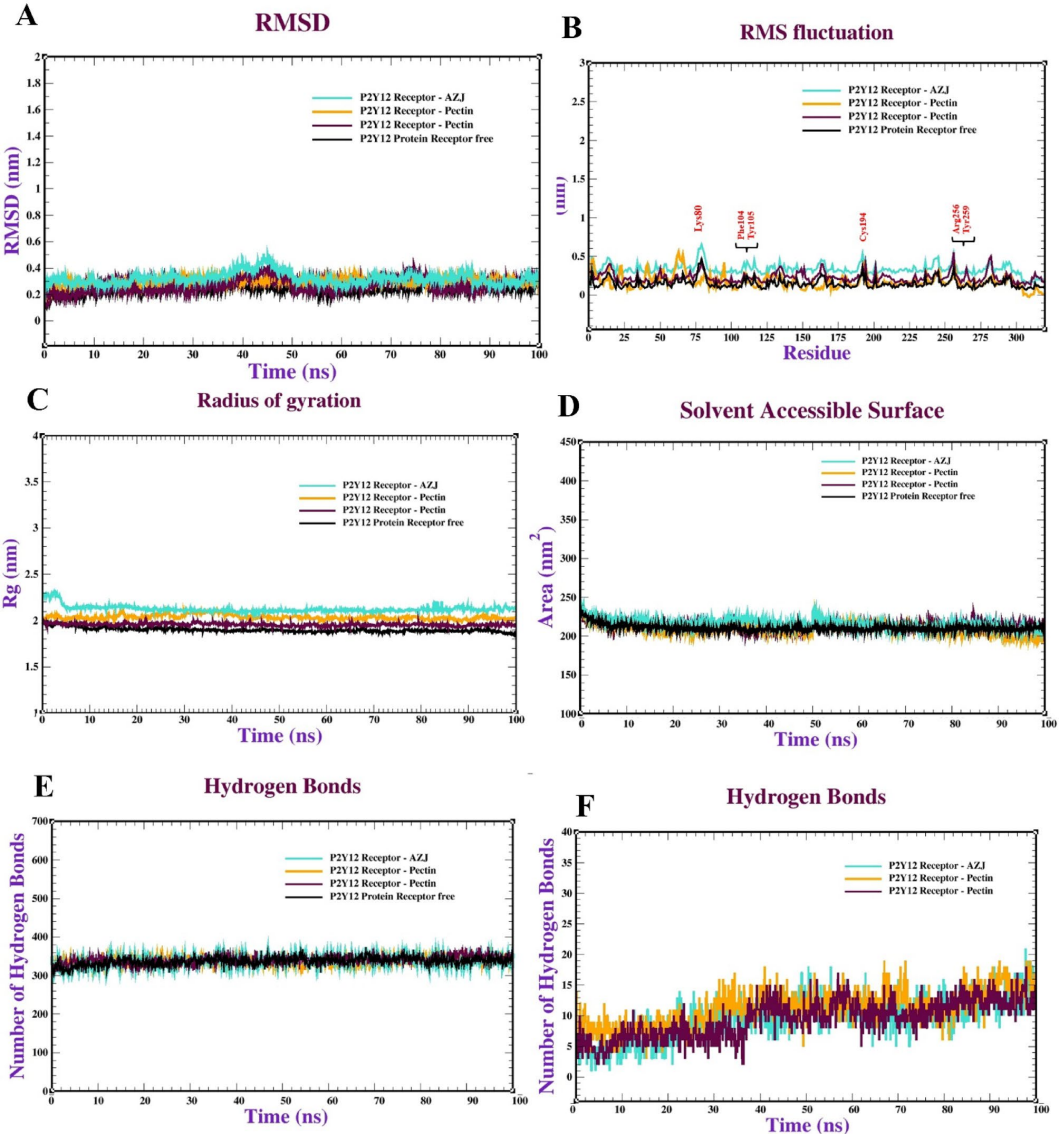
Molecular dynamics of the P2Y12 receptor (PDB: ID 4PXZ): complexed with pectin: (**a**) RMSD, (**b**) RMSF, (**c**) radius of gyration (R_g_), (**d**) SASA, (**e**) intramolecular hydrogen bonds and (**f**) intermolecular hydrogen bonds.

The Pectin-P2Y12 complex (Fig. 7) exhibits poor stability, consistent with its docking performance. The RMSD (panel a) fluctuates significantly (~ 0.25–0.3 nm, 2.5–3 Å) with peaks up to 0.31 nm, indicating instability and potential ligand repositioning (docking RMSD: 1.05 Å). RMSF (panel b) shows low fluctuations (< 0.2 nm) with loop peaks (~ 0.3 nm), similar to other complexes. SASA (panel c) varies between 140 and 160 nm², and Rg (panel d) fluctuates at 2.45–2.5 nm, suggesting conformational changes and reduced compactness. Intramolecular H-bonds (panel e) are stable (~ 300–340), but intermolecular H-bonds (panel f) are minimal (0–2, often 0), aligning with docking’s 5 H-bonds and low affinity (-4.70 kcal/mol). Pectin’s weak performance underscores its unsuitability as a standalone ligand, though it contributes to the dual-ligand’s efficacy. Table 7 tracks RMSD values at 0–100 ns, confirming the dual-ligand’s superior stability. The 2-Oleu(OH)-Pec(OH) complex stabilizes at ~ 0.414 nm (4.14 Å) from 10 ns onward with minimal fluctuations, aligning with Fig. 5 and docking’s low RMSD (0.68 Å). Oleuropein’s RMSD rises to 0.30811 nm (3.08 Å), and AZJ reaches 0.33451 nm (3.35 Å), indicating moderate stability (docking RMSDs: 0.88 Å and 0.65 Å). Pectin’s RMSD fluctuates (0.25109 nm at 100 ns, peak at 0.31008 nm), reflecting instability (docking RMSD: 1.05 Å). The consistent RMSD trend (dual-ligand > AZJ > Oleuropein > Pectin) validates the docking stability rankings, emphasizing the dual-ligand’s robust binding dynamics.

Additionally, The MM/PBSA analysis (Table 8) quantifies the binding free energy (ΔG) of the P2Y12-ligand complexes, reinforcing the docking results. The dual-ligand 2-Oleu(OH)-Pec(OH) achieves the lowest ΔG (-54.64 ± 5.30 kcal/mol), driven by strong van der Waals (-35.51 kcal/mol) and non-polar interactions (– 40.14 kcal/mol), surpassing Oleuropein (-48.55 ± 3.55 kcal/mol) and Pectin (-44.55 ± 6.22 kcal/mol). AZJ’s ΔG (-52.50 ± 4.33 kcal/mol) is slightly higher than the dual-ligand’s, despite stronger van der Waals energy (-41.00 kcal/mol). Pectin’s poor ΔG reflects its weak interactions, consistent with docking’s low affinity (-4.82 kcal/mol) and minimal bonds (7). The dual-ligand’s favorable ΔG correlates with its docking affinity (-6.76 kcal/mol) and extensive interactions (18 bonds), highlighting its thermodynamic stability and potential as a P2Y12 inhibitor. Also, the MM/PBSA results reinforce the docking findings, with the 2-Oleu(OH)-Pec(OH) low ΔG (-54.64 kcal/mol) reflecting its strong binding affinity and stability. The dominance of van der Waals and non-polar interactions in all complexes highlights the importance of hydrophobic contacts in P2Y12 binding, consistent with docking’s Pi-Alkyl and Carbon-H bonds.

**Fig. 8:**
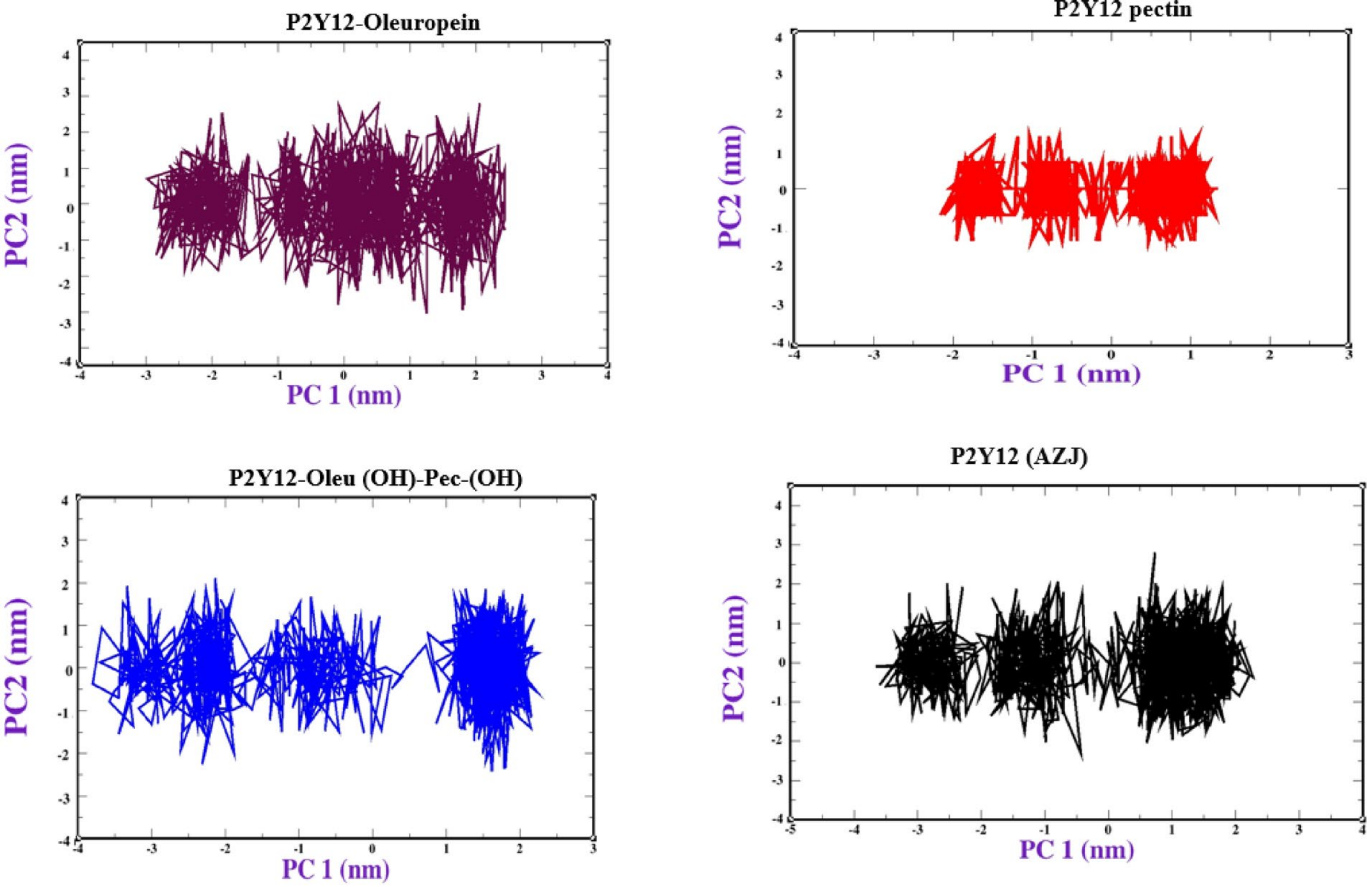
Principal component analysis of P2Y12 receptor complexes.

Moreover, The Principal Component Analysis (PCA) of the P2Y12 receptor (PDB ID: 4PXZ) complexed with four ligands reveals distinct conformational dynamics (Fig. 8). The 2-Oleu(OH)-Pec(OH) complex exhibits the tightest clustering, with data points spanning − 2 to 1 nm along PC1 and − 2 to 1 nm along PC2, indicating minimal conformational variability and high stability. This aligns with its docking RMSD (0.68 Å), MD RMSD (~ 0.41 nm, Table 8), and favorable MM/PBSA ΔG (-54.64 kcal/mol, Table 7), driven by extensive interactions (12 H-bonds, 18 total bonds in docking; 2–4 H-bonds in MD, Fig. 4f). AZJ shows a moderate compact cluster (-3 to 2 nm on PC1, -2 to 2 nm on PC2), reflecting good stability, consistent with its docking RMSD (0.65 Å) and MD ΔG (-52.50 kcal/mol). Oleuropein’s cluster (-2 to 2 nm on PC1, -3 to 2 nm on PC2) is similar to AZJ’s, indicating moderate stability (docking RMSD: 0.88 Å, MD ΔG: -48.55 kcal/mol). Pectin displays the widest spread (-3 to 2 nm on PC1, -2 to 2 nm on PC2), suggesting high flexibility and lower stability, which correlates with its high docking RMSD (1.05 Å), fluctuating MD RMSD (0.25–0.31 nm), and poor ΔG (-44.55 kcal/mol). The PCA stability order (dual-ligand > AZJ > Oleuropein > Pectin) matches the docking and MD trends, highlighting the dual-ligand’s ability to constrain receptor motions, likely due to its synergistic binding interactions, making it a promising candidate for P2Y12 inhibition. Our molecular dynamics simulations aligned with the findings of Halim et al.,^44^ who utilized a comparable methodology to investigate drug-like molecules targeting the P2Y12 receptor protein. In summary, The MD simulation results strongly support the docking findings, confirming that the dual-ligand 2-Oleu(OH)-Pec(OH) is a highly stable and potent P2Y12 receptor binder, with a low RMSD (~ 0.41 nm), favorable binding free energy (-54.64 kcal/mol), and robust H-bonding (2–4 intermolecular H-bonds). It outperforms Oleuropein and Pectin, rivaling the native ligand AZJ, due to synergistic interactions between Oleuropein and Pectin. Pectin’s poor stability and weak binding (high RMSD, low ΔG) highlight its limitations as a standalone ligand, though it enhances the dual-ligand’s performance. The consistent receptor flexibility (RMSF) and internal stability (intramolecular H-bonds) across complexes underscore ligand-specific differences in binding efficacy. These results suggest that 2-Oleu(OH)-Pec(OH) is a promising candidate for P2Y12 inhibition, warranting further experimental validation through in vitro assays or extended MD simulations.

**Table 7:** The RMSD values (nm units) of the P2Y12 -ligand complex system recorded at various time intervals (0, 20, 40, 60, 80 & 100 ns).

P2Y12 -ligand complex	Time (ns)						
	0 ns	10 ns	20 ns	40 ns	60 ns	80 ns	100ns
2- Oleu (OH)-Pec (OH)	0.0081	0.41510	0.40551	0.413321	0.41384	0.414510	0.41413
Oleuropein	0.001	0.21010	0.24310	0.2841	0.30510	0.30610	0.30811
Pectin	0.1110	0.24100	0.25881	0.31008	0.28410	0.25101	0.25109
AZJ	0.005	0.31110	0.3210	0.45121	0.3510	0.33010	0.33451

**Table 8:** Summary of MM/PBSA free energy Estimation of P2Y12 complexes.

System	Δ Electrostatic energy	Δ Van der Waals energy	Δ Poisson Boltzmann	Δ Surface area	Δ Gas phase energy	Δ Solvation free energy	Δ Polar interactions	Δ Non-polar interactions	Total free energy ΔG
2- Oleu (OH)-Pec (OH)	− 2.27 ± 0.20	− 35.51 ± 2.05	28.80 ± 4.22	− 8.50 ± 0.32	− 36.79 ± 3.64	25.28 ± 4.30	14.50 ± 1.30	− 40.14 ± 2.70	− 54.64 ± 5.30
Oleuropein	−1.55 ± 0.89	−28.50 ± 1.80	19.80 ± 3.00	− 7.52 ± 0.10	− 36.79 ± 3.64	22.80 ± 5.22	12.56 ± 1.02	− 35.50 ± 5.66	− 48.55 ± 3.55
Pectin	-1.52 ± 0.54	−26.40 ± 1.80	22.30 ± 2.55	− 4.63 ± 0.44	− 36.79 ± 3.64	15.55 ± 1.22	10.55 ± 0.81	− 28.90 ± 3.55	− 44.55 ± 6.22
AZJ	−2.40 ± 0.35	−41.00 ± 2.40	25.75 ± 3.55	− 9.51 ± 0.30	− 36.79 ± 3.64	22.90 ± 1.32	16.88 ± 0.55	− 38.55 ± 1.35	− 52.50 ± 4.33

## Conclusion

This study breaks new ground by integrating natural polymer chemistry with advanced computational modeling to redefine Oleuropein’s therapeutic applicability. Unlike conventional P2Y12 inhibitors, which rely on irreversible covalent binding and carry bleeding risks, the Oleu-Pec complex exploits reversible physical interactions via OH groups—a mechanism not yet explored in antiplatelet drug design. Molecular modelling concepts were carried out to study the feasibility of modifying Oleuropein structure with pectin biomolecule to improve its hydrophilicity and absorption in the gut aqueous media. Model molecules of Oleuropein, pectin and some proposed interactions were built-up and geometrically optimized via both semiempirical and DFT high theoretical levels. The geometries were first optimized at PM6 method to calculate the QSAR descriptors which ascertain the ability of pectin structure to improve hydrophilicity of Oleuropein structure with negative partition coefficient values (− 1.171). Then, the structures were reoptimized using B3LYP/6-311 G (d, p) model chemistry. DFT calculations reveal that Oleu-Pec proposed structures have stable characteristics and chemical reactivity with dipole moment up to 7.86 Debye. They also indicate that the interaction between Oleu and Pec structures is most probable to occur between the OH of Pec and that of Oleu either in the glucose moiety or on the other side of Oleu structure. MESP maps were conducted to reveal the active sites in the proposed structures. These configurations can be correlated with the presence of the tendency of the proposed structures to go through both nucleophilic and electrophilic pathways. Furthermore, global reactivity descriptors were calculated to reveal the improved electronic features of Oleu-Pec structures upon the addition of Pec to Oleu proposing it for further promising biomedical applications. The combined docking and molecular dynamics (MD) simulations of the P2Y12 receptor with ligands 2-Oleu(OH)-Pec(OH), Oleuropein, Pectin, and AZJ reveal significant insights into their binding behavior. The dual-ligand 2-Oleu(OH)-Pec(OH) emerges as the most promising candidate, exhibiting the highest stability with a docking RMSD of 0.68 Å, a strong binding affinity (-6.80 kcal/mol), and an extensive interaction network (18 total bonds, 12 H-bonds). MD simulations confirm its stability with a consistent RMSD (~ 0.41 nm), 2–4 intermolecular H-bonds, and the lowest MM/PBSA ΔG (− 54.64 kcal/mol). Principal Component Analysis (PCA) further supports this, showing the tightest clustering, indicating minimal conformational flexibility. The synergy between Oleuropein and Pectin in the dual-ligand enhances binding strength, surpassing individual components. These results highlight the 2-Oleu(OH)-Pec(OH) potential as a P2Y12 inhibitor for anti-thrombotic applications. Also, this work establishes a blueprint for leveraging natural polymer interactions in drug design, addressing both efficacy and tolerability a critical gap in current computational pharmacology.

## Data Availability

The data and materials are available from the corresponding author upon request.
